# Calcium homeostasis in aging neurons

**DOI:** 10.3389/fgene.2012.00200

**Published:** 2012-10-02

**Authors:** Vassiliki Nikoletopoulou, Nektarios Tavernarakis

**Affiliations:** Institute of Molecular Biology and Biotechnology, Foundation for Research and Technology – HellasHeraklion, Crete, Greece

**Keywords:** endoplasmic reticulum, Golgi, long-term potentiation, ion channel, mitochondria, neurodegeneration, neurotransmitter, synaptic plasticity

## Abstract

The nervous system becomes increasingly vulnerable to insults and prone to dysfunction during aging. Age-related decline of neuronal function is manifested by the late onset of many neurodegenerative disorders, as well as by reduced signaling and processing capacity of individual neuron populations. Recent findings indicate that impairment of Ca^2+^ homeostasis underlies the increased susceptibility of neurons to damage, associated with the aging process. However, the impact of aging on Ca^2+^ homeostasis in neurons remains largely unknown. Here, we survey the molecular mechanisms that mediate neuronal Ca^2+^ homeostasis and discuss the impact of aging on their efficacy. To address the question of how aging impinges on Ca^2+^ homeostasis, we consider potential nodes through which mechanisms regulating Ca^2+^ levels interface with molecular pathways known to influence the process of aging and senescent decline. Delineation of this crosstalk would facilitate the development of interventions aiming to fortify neurons against age-associated functional deterioration and death by augmenting Ca^2+^ homeostasis.

## INTRODUCTION

Fluctuations in intracellular calcium concentration act as signals for a variety of processes in neurons. Most notably, Ca^2+^ is the major trigger of neurotransmitter release, a process that has been thoroughly investigated over the past decades (Neher and Sakaba, 2008). Moreover, it has also become clear that Ca^2+^ is essential for a variety of other neuronal functions, including neuronal excitability (Marty and Zimmerberg, 1989), integration of electrical signals (Llinas, 1988; Marty and Zimmerberg, 1989), synaptic plasticity (Malenka et al., 1989), gene expression (Szekely et al., 1990), metabolism (McCormack and Denton, 1990), and programmed cell death (Chalfie and Wolinsky, 1990). Given its central role in processes that are fundamental to the excitable nature of neurons, Ca^2+^ homeostasis is tightly regulated in these cells (see **Table 1** for a summary of the key effectors of Ca^2+^ homeostasis, in neurons). Here, we briefly overview the main mechanisms neurons use in order to achieve an intricate regulation of the intracellular concentration of Ca^2+^. In addition, we discuss the accumulating evidence on the potential role of deregulated Ca^2+^ homeostasis in aging and disease of the nervous system.

**Table 1:** Summary of different Ca^2+^ channels, buffers and sensors, their subcellular localization and function.

	Sub-cellular localization	Function
Channels
Voltage-gated Ca^2+^ channels NMDA receptor	Plasma membrane	Influx of Ca^2+^ into the cell
PMCA, ATP driven ca^2+^ pump NCX, “Na+/ca^2+^ exchanger”		Efflux of ca^2+^ from the cell
SERCA 1, 2a, 2b, 3	ER and Golgi	Influx of ca^2+^ into the ER or Golgi
Inositol 3-phosphate (InsP3) receptors	ER	Efflux of ca^2+^ from the ER
Ryanodine receptors (RyRs)		
NAADP receptors		
polycystin-2 channels		
presenilin 1 and 2		
SPCA 1a, 1b, 1c, 1d, 2	Golgi	Influx of ca^2+^ into the GolgiX
ca^2+^ uniporter	Mitochondria	Influx of ca^2+^ into mitochondria
NCX mitochondrial Na+/ca^2+^ exchanger mPTP		Efflux of ca^2+^ from mitochondria
Buffers
Calreticulin	ER	Reversible sequestering of ca^2+^
Calsequestrin		
Endoplasmin		
BiP/grp78		
Reticulocalbin		
CREC family proteins		
Calretinin	Cytosol, mainly CNS GABAergic interneurons	
Calbindin		
Parvalbumin		
Nucleo-calbindin	Golgi	
Glycerophosphate dehydrogenase Aralar ARE	Mitochondrial	
Sensors
Calmodulin	Cytosol	Translation of graded ca^2+^ concentration changes into graded signaling responses via interaction with ca^2+^ sensitive enzymes
Recoverins	Cytosol, photoreceptors	
Guanylyl cyclase activating protein 1 (GCAP1) Frequenins	Cytosol, CNS neurons	Kv channel interacting proteins (KChIPs)

## MECHANISMS OF NEURONAL CALCIUM HOMEOSTASIS RELEVANT TO AGING AND DEGENERATION

### Ca^2+^ INFLUX THROUGH THE PLASMA MEMBRANE

**FIGURE 1:**
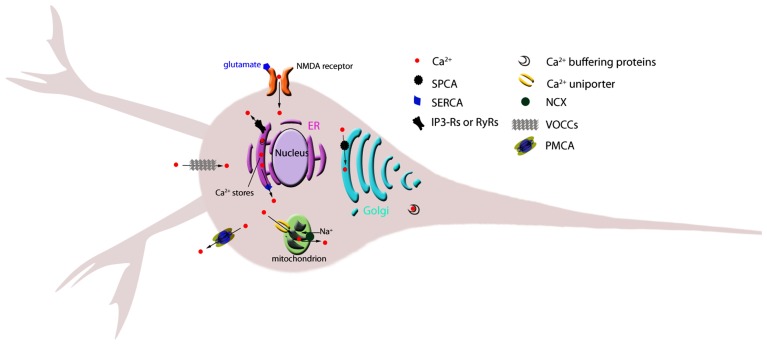
** Schematic representation of the main Ca^2+^ homeostatic machinery components in neurons.** Individual, key components of calcium homeostatic mechanisms discussed in the text are shown. Arrows indicate direction of ion flux. ER, endoplasmic reticulum; IP3-R, inositol 3-phosphate receptor; NCX, sodium calcium exchanger; NMDA,* N*-methyl-D-aspartate; PMCA, plasma membrane Ca^2+^ ATPase; RyR, ryanodine receptor; SERCA, sarco(endo)plasmic reticulum Ca^2+^ ATPase; SPCA, secretory-pathway Ca^2+^-ATPase; VOCC, voltage-operated calcium channel.

Plasma membrane Ca^2+^ channels allow the passive influx of calcium ions down their electrochemical gradient. These channels are categorized into two major groups depending on the mechanism controlling their transition between the open and closed conformations: channels gated by voltage (also known as voltage-operated Ca^2+^ channels, VOCC), and channels gated by ligand binding, in neurons usually L-glutamate (**Figure 1**; **Table 1**).

Voltage-gated Ca^2+^ channels are multi-protein complexes comprising several different subunits: α_1_, α_2_δ, β_1__-__4_, and γ (Takahashi and Catterall, 1987; Catterall et al., 1990). The α_1_ subunit is the largest and it contains the conduction pore, the voltage sensors, and gating apparatus, and most of the known sites of channel regulation by second messengers, drugs, and toxins. The α_1_ subunits are associated with distinct auxiliary protein subunits (Catterall et al., 1990): the intracellular β subunit, the transmembrane, disulfide-linked α_2_δ subunit complex, and the γ subunit, a component of skeletal muscle Ca^2+^ channels also expressed in heart and brain having four transmembrane segments. Although these auxiliary subunits modulate the functional properties of the Ca^2+^ channel complex, the pharmacological and physiological diversity of Ca^2+^ channels arises primarily from the existence of multiple α_1_ subunits. These are encoded by 10 distinct genes in mammals, further divided into three subfamilies based on sequence similarity (Catterall et al., 1990; Snutch and Reiner, 1992; Ertel et al., 2000). Division of Ca^2+^ channels into these three subfamilies is phylogenetically ancient, as single representatives of each are found in the *Caenorhabditis elegans* genome. Recently, calcium homeostasis modulator 1 (CALHM1), a glycosylated membrane protein expressed throughout the brain, was identified as the pore-forming subunit of a unique plasma membrane Ca^2+^-permeable voltage-gated ion channel (Ma et al., 2012).

Based on the characteristics of channel composition, distinct classes of Ca^2+^ currents have been described (Tsien et al., 1988). In summary, N-type, P/Q-type, and R-type Ca^2+^ currents are induced upon strong depolarization (Tsien et al., 1991) and are pharmacologically blocked by specific toxins derived from snail and spider venoms (Miljanich and Ramachandran, 1995). N-type and P/Q-type Ca^2+^ currents are observed primarily in neurons where they initiate neurotransmission at most fast conventional synapses (Catterall et al., 1990; Olivera et al., 1994; Dunlap et al., 1995). More specifically, the CaV2 subfamily members (CaV2.1, CaV2.2, and CaV2.3) conduct P/Q-type, N-type, and R-type Ca^2+^ currents, respectively (Catterall et al., 1990; Snutch and Reiner, 1992; Olivera et al., 1994; Ertel et al., 2000). Ca^2+^ entering neurons through the CaV2.1 and CaV2.2 channels is primarily responsible for initiating synaptic transmission at conventional fast synapses (Olivera et al., 1994; Dunlap et al., 1995). CaV2.2 channels are most prevalent at synapses formed by neurons of the peripheral nervous system. In contrast, CaV2.1 channels play a major role at most synapses formed by neurons of the mammalian central nervous system. However, in some central synapses, including a subset of inhibitory interneurons of the hippocampus (Poncer et al., 1997), CaV2.2 channels are predominant in neurotransmitter release.

Ca^2+^ entry through a voltage-gated Ca^2+^ channel initiates neurotransmission by triggering vesicular release (Stanley, 1993). Ca^2+^-triggered synaptic vesicle exocytosis depends on the assembly of the SNARE complex, in which the vesicle-associated v-SNARE protein synaptobrevin (VAMP) interacts with two plasma membrane-associated t-SNARE proteins, SNAP-25 and syntaxin-1 (Sollner et al., 1993; Bajjalieh and Scheller, 1995; Sudhof, 1995,^2004^). Maturation into a release-ready SNARE complex requires synaptotagmin, an integral Ca^2+^-binding protein of the synaptic vesicle membrane that provides Ca^2+^-dependent regulation of the fusion machinery. Ca^2+^ influx into the presynaptic terminal binds to the Ca^2+^ sensor, synaptotagmin, and the SNARE complex changes conformation from a *trans *to a *cis* state, resulting in the fusion of apposing membranes and the release of neurotransmitter. Neurotransmitter release occurs in two phases: a fast synchronous (phasic) component and a slow asynchronous (tonic) component (Hubbard, 1963; Barrett and Stevens, 1972; Rahamimoff and Yaari, 1973; Goda and Stevens, 1994; Atluri and Regehr, 1998). Both forms of transmission are Ca^2+^ dependent. Synchronous release driven by the precisely timed presynaptic Ca^2+^ current results in a large, fast postsynaptic response (Llinas et al., 1981; Sabatini and Regehr, 1996), whereas the slower asynchronous component, resulting from residual Ca^2+^ remaining in the terminal after an action potential, provides a basal or tonic level of neurotransmitter release at many synapses (Atluri and Regehr, 1998; Lu and Trussell, 2000; Hagler and Goda, 2001).

In addition to voltage-gated channels, a number of Ca^2+^ channels on the plasma membrane of neurons are activated by the interaction of ligands with their own plasma membrane receptors. The most prominent such ligand in the nervous system is L-glutamate, by far the most widespread excitatory transmitter in the vertebrate central nervous system. L-glutamate activates two general classes of receptors, the “ionotropic” receptors, which are ionic channels, and the G-protein coupled “metabotropic” receptors. Of these, the ionotropic receptors mediate the direct penetration of Ca^2+^ into the cell. Three forms of ionotropic receptors have been characterized and named after their most widely used agonists. These are the kainate (KA) receptors, the α-amino-3-hydroxy-5-methyl-4-isoxazole propionate (AMPA) receptors, and the *N*-methyl-D-aspartate (NMDA) receptors. The channels formed by AMPA and KA receptors are primarily permeable to Na^+^ and K^+^ and exhibit a rather low conductance to Ca^2+^ (Mayer and Westbrook, 1987). By contrast, the NMDA receptors have a considerably higher conductance and are permeable to Na^+^ and Ca^2+^ (MacDermott et al., 1986). These receptors do not mediate rapid synaptic transmission, their contribution being primarily to the slow component of excitatory postsynaptic currents. At the resting plasma membrane potential they are powerfully inhibited by Mg^2+^, whose block is reversed by plasma membrane depolarization (Nowak et al., 1984). Thus, the rapid increase of membrane depolarization following the activation of KA/AMPA receptors by glutamate released into the synaptic cleft reduces the inhibition of NMDA receptors by Mg^2+^. Therefore, the excitatory postsynaptic potential produced by activation of an NMDA receptor highly increases the concentration of Ca^2+^ in the cell. The Ca^2+^ in turn functions as a key second messenger in various signaling pathways. The ability of the NMDA receptor to act as a “coincidence receptor,” requiring the concomitant presence of its ligand and membrane depolarization in order to be activated, explains many aspects of its functional involvement in long-term potentiation (LTP) and synaptic plasticity, a process associated with memory and learning as discussed later.

### EFFLUX OF CALCIUM THROUGH THE PLASMA MEMBRANE

Two major plasma membrane mechanisms are responsible for the extrusion of Ca^2+^ from cells (**Figure 1**; **Table 1**). One is the ATP-driven plasma membrane Ca^2+^ pump (PMCA) and the other is the Na^+^/Ca^2+^ exchanger (NCX), a complex similar to that discussed later for the removal of Ca^2+^ from the mitochondrial matrix into the cytoplasm (Baker and Allen, 1984; Carafoli and Longoni, 1987; Blaustein, 1988). Unlike in mitochondria, plasma membrane NCX has the inherent ability to move Ca^2+^ into or out of the cell depending on the prevailing conditions. When the system is acting to remove Ca^2+^, energy is supplied by the electrochemical gradient that ultimately results from the activity of the plasma membrane Na^+^/K^+^ ATPase (Na^+^ pump).

Plasma membrane Ca^2+^ pump has a higher affinity for Ca^2+^ (*K*_d_ = 100 nM) but a very slow turnover, whereas NCX has a much lower affinity (*K*_d_ = 1000 nM) but a higher turnover. Both types of transporters are co-expressed in neurons and in astrocytes (DiPolo and Beauge, 1983; Juhaszova et al., 2000). However, the precise role that each plays in removing excess Ca^2+^ loads under different physiological and pathophysiological conditions remains rather unclear. A major difference is the fact that they exhibit distinct subcellular localization patterns. In particular, some if not all of PMCA found in neurons seems to be localized very close to the neurotransmitter release sites (active zone) of the presynaptic terminals, whereas NCX is excluded from these sites and present in a more dispersed fashion on the rest of the neuron (Juhaszova et al., 2000; Blaustein et al., 2002). Therefore, the PMCA may help keep active zone Ca^2+^ very low, and function to re-prime the neurotransmitter release mechanism following activity. NCX, on the other hand, is believed to efflux Ca^2+^ that has diffused away from the active zone and perhaps been temporarily sequestered by the endoplasmic reticulum (ER). Moreover, the discovery of a multitude of PMCA isoforms and alternative splice variants (Strehler and Treiman, 2004; Strehler et al., 2007), as well as recent results on PMCA “knockout” mice and PMCA mutants (Prasad et al., 2007), show that at least some PMCAs play a more specific role in local Ca^2+^ handling. In addition, a growing number of specific PMCA-interacting proteins have been identified with regulatory, targeting, and signaling functions. These findings support a new paradigm, whereby PMCAs are not only responsible for global Ca^2+^ homeostasis but are dynamic participants in spatially defined Ca^2+^ signaling. The main regulator of PMCA function is Ca^2+^ calmodulin (Ca^2+^-CaM; Werth et al., 1996). In the absence of CaM, the pumps are autoinhibited by a mechanism that involves the binding of their C-terminal tail to the two major intracellular loops. Activation requires binding of Ca^2+^-CaM to the C-terminal tail and a conformational change that displaces the autoinhibitory tail from the major catalytic domain. Release of autoinhibition may be facilitated by means other than CaM binding, including by acidic phospholipids, protein kinase A- or C-mediated phosphorylation of specific (Ser/Thr) residues in the C-terminal tail (Werth et al., 1996), partial proteolytic cleavage of the tail (e.g., by calpain or caspases), or dimerization via the C-terminal tail (for a detailed review see Di Leva et al., 2008). Different PMCA isoforms show significant differences in their regulation by kinases and CaM. Interestingly, loss of PMCA function was reported to lead to an increase in the levels of intracellular Ca^2+^, causing apoptotic death of cerebellar and spinal cord neurons (Kurnellas et al., 2007).

### INTRACELLULAR CALCIUM HOMEOSTASIS IN NEURONS

#### Ca^2+^homeostasis in the ER

The ER, a complex system of endomembranes, is present in all neurons and extends from the nucleus to the soma, dendrites, and dendritic spines, and down the axon to the presynaptic terminals. Particularly relevant for neuronal function is the ability of the ER to act as a dynamic Ca^2+^ store, able to actively accumulate Ca^2+^ and to release it in response to physiological stimulation. As such, the ER contains a variety of channels, buffers, and sensors dedicated to Ca^2+^ homeostasis (**Figure 1**; **Table 1**). In general, Ca^2+^ exits the ER through several types of Ca^2+^ release channels, such as inositol 3-phosphate (InsP3) receptors, ryanodine receptors (RyR), nicotinic acid adenine dinucleotide phosphate (NAADP) receptors, and polycystin-2 channels [the relative of transient receptor potential (TRP) proteins]. In neurons, the NAADP receptors were reported to exist in brain microsome preparations (Bak et al., 1999) and Ca^2+^ release from these channels was described in neurons from the buccal ganglion of aplysia (Chameau et al., 2001), yet their relevance in vertebrate neurons remains unclear. Regarding the TRPs, although they are expressed by neurons, there is so far no evidence for their involvement in Ca^2+^ homeostasis in these cells. Therefore, in neurons, Ca^2+^ exit from the ER occurs mainly through the inositol 3-phosphate receptors (IP3-Rs) and the Ca^2+^ activated RyR, both forming large tetrameric channel proteins. Both receptor families are comprised of multiple members that display distribution patterns that are both temporally and spatially regulated in neurons. For example, there are three RyRs, all of which can be activated by Ca^2+^ on the cytosolic side with differential sensitivities (RyR1 > RyR2 > RyR3). All three members have been detected in neurons, with distinct patterns that change during development and postnatal growth. For example, postnatally, RyR1 is highly expressed in cerebellar Purkinje cells, RyR3 in the hippocampus, striatum, and diencephalon, while many neurons co-express more than one RyR isoform (Hakamata et al., 1992; Lai et al., 1992; Furuichi et al., 1994; for review also see Berridge, 1998; Hertle and Yeckel, 2007). Regarding their sub-cellular localization, RyRs have been seen in all parts of neurons, including the soma, axons, dendrites, and even the spine apparatus of excitatory neurons. Similarly, there are three InsP3R isoforms with different sensitivities to Ca^2+^, and further diversity may arise from alternative splicing of InsP3R1. InsP3R1 is the main isoform in neurons in the brain, while InsP3R3 is mainly found in the spinal cord and in glial cells (Berridge, 1998).

Propagating Ca^2+^ waves is the most dramatic expression of Ca^2+^ release from the ER, reflecting the Ca^2+^-induced Ca^2+^ release (CICR) mode, where elevated cytoplasmic Ca^2+^ induces further Ca^2+^ release. Ca^2+^ waves in neurons were described more recently, after the notion had first been established using *Xenopus* oocytes (Lechleiter et al., 1991; Parker and Ivorra, 1991). Given the functional compartmentalization of neurons, Ca^2+^ waves take up different properties depending on their spatial localization and neuronal type diversity. For example, synaptically activated Ca^2+^ waves preferentially initiate at branch points of dendrites (Nakamura et al., 2002; Larkum et al., 2003; Fitzpatrick et al., 2009) and are mediated by the IP3-Rs (Nakamura et al., 1999). Such waves have been observed in pyramidal neurons of the rodent CA1 and CA3 regions of the hippocampus (Miller et al., 1996; Kapur et al., 2001), in layers 2 and 3 of the cortex (Larkum et al., 2003; Hagenston et al., 2008) and principal neurons of the amygdala (Power and Sah, 2008), all regions heavily involved in memory and learning. Relevant to the cognitive decline and memory loss associated with aging, synaptically induced Ca^2+^ waves are functionally linked to synaptic plasticity, a process known to require a rise in the postsynaptic concentration of Ca^2+^. More specifically, there are several cases where synaptically activated Ca^2+^ release from stores was shown to induce LTP (Yeckel et al., 1999), though it remains controversial as one study challenged this conclusion (Mellor and Nicoll, 2001).

In addition to the channels discussed above, some studies have suggested that presenilin 1 and 2, beyond constituting the proteases in the γ-secretase complex, also function as Ca^2+^ leak channels in the ER, either by themselves, or indirectly by increasing the activity of IP3-Rs and RyRs (Pack-Chung et al., 2000). In particular, presenilin 2 was shown to interact with sorcin, a cytoplasmic calcium-binding protein that modulates the activity of RyRs (Pack-Chung et al., 2000). Interestingly, in some mutations of presenilin 1 and 2 that are responsible for familial Alzheimer’s disease, disruption of intracellular Ca^2+^ homeostasis by the ER is the major measurable cellular consequence (Nelson et al., 2010), as discussed later on.

Calcium uptake into the ER lumen results from the function of Ca^2+^ pumps of the P-type sarco(endo)plasmic reticulum Ca^2+^ ATPase (SERCA) family. This family includes three members (SERCA1–3), as well as two splice isoforms of SERCA2. While SERCA2b is ubiquitously expressed, SERCA2a and SERCA3 are found almost exclusively in cerebellar Purkinje neurons. Inhibition of the SERCA pumps results in a relatively slow emptying of ER Ca^2+^ stores, with Ca^2+^ exiting the ER through poorly described pathways (Camello et al., 2002). Ca^2+^ buffering in the ER lumen is achieved by specific Ca^2+^-binding proteins. In neurons, the most abundant of these is calreticulin and calsequestrin, while some others such as endoplasmin, BiP/grp78, and proteins of the CREC family also participate in Ca^2+^ buffering. A main difference of ER Ca^2+^ buffers is that, unlike their cytosolic counterparts, they have a low affinity for Ca^2+^ to allow the maintenance of high intra-ER Ca^2+^ levels.

#### Ca^2+^ homeostasis in the Golgi

Ca^2+^ uptake in the Golgi apparatus involves two groups of Ca^2+^ pumps: the well characterized SERCAs, discussed above, and a less characterized group of ATPases that were described as secretory-pathway Ca^2+^-ATPases (SPCAs; Shull, 2000; **Figure 1**; **Table 1**). The SPCAs in addition supply the Golgi lumen with Mn^2+^, which is needed for many enzymatic reactions in this compartment. Mammalian SPCA was originally cloned from rat using a probe derived from sequences of the ATP-binding site of SERCA1 and SERCA2 (Gunteski-Hamblin et al., 1992). The corresponding human gene (*ATP2C1*) was described by two independent groups (Hu et al., 2000; Sudbrak et al., 2000). Alternative processing of *ATP2C1* results in four SPCA1 proteins with C-termini differing in length and specific amino acid sequence (Hu et al., 2000; Sudbrak et al., 2000; Fairclough et al., 2003), SPCA1a, SPCA1b, SPCA1c, and SPCA1d. Ishikawa et al. (1998) later described a second human SPCA isoform, named SPCA2. Its human gene (*ATP2C2*) was independently described in 2005 by two groups (Vanoevelen et al., 2005; Xiang et al., 2005). The widespread expression pattern of SPCA1 and the observation that homozygous loss of a functional *ATP2C1* gene do not seem to be viable suggest that SPCA1 is a housekeeping enzyme. The tissue and cellular expression of SPCA2 appears to be more restricted than that of SPCA1, and based on mRNA data it is expressed in the brain, among other tissues (Vanoevelen et al., 2005; Xiang et al., 2005). It is now well established using a range of different cell types that the endogenous SPCA1 is specifically located in the Golgi compartment (Behne et al., 2003; Van Baelen et al., 2003; Reinhardt et al., 2004; Ramos-Castaneda et al., 2005). The relative contribution of SERCAs and SPCAs to the total uptake of Ca^2+^ into the Golgi apparatus seems to be cell-type-dependent. The highest dependence on SPCAs occurs in human keratinocytes (Callewaert et al., 2003). This finding is important for explaining the physiopathology of the skin-related Hailey–Hailey disease.

While the potentially specific roles of SPCAs in neurons are poorly understood, our own recent findings (Kourtis et al., 2012) suggest that SPCA1 is both necessary and sufficient in mediating the neuroprotective function of heat preconditioning in a model of heat stroke-induced neurodegeneration. Notably, this mechanism is evolutionarily conserved as it is preserved from *C. elegans* to mammals. This finding invites the speculation that SPCAs may have a more general neuroprotective role, whose relevance to other forms of neurodegeneration and aging remains to be examined.

#### Ca^2+^ homeostasis by mitochondria

Beyond their main role in the cell to produce NADH and ATP, it is now well accepted that mitochondria also function as Ca^2+^ buffers (**Figure 1**; Table 1). As proton pumping creates an inside-negative membrane potential in mitochondria, Ca^2+^ tends to be drawn into the mitochondrial matrix following its electrochemical gradient. This influx is mainly achieved by the mitochondrial Ca^2+^ uniporter whose conductance is dependent on both intracellular Ca^2+^ concentration and energy demand. At high cytosolic Ca^2+^ concentrations and low ATP/ADP ratio more Ca^2+^ is conducted, whereas at low cytosolic Ca^2+^ concentration and high ATP/ADP ratio less Ca^2+^ is conducted. Intricately enough, increasing mitochondrial Ca^2+^ concentration activates the enzymes of the Krebs cycle, thus causing increased ATP production. As mitochondrial Ca^2+^ buffering is more energy efficient compared to expelling Ca^2+^ through the plasma membrane or into the ER, this mechanism is considered of high relevance for neurons in situations when ATP and oxygen demands reach high levels, such as in the case of repeated axon potentials (Contreras et al., 2010).

Calcium is expelled from the mitochondrial matrix into the cytosol mainly by the mitochondrial sodium calcium exchanger (NCX; three Na^+^ for one Ca^2+^), in conditions of low ATP demand and oxygen consumption, or through a mitochondrial proton/Ca^2+^ exchanger (two or more H^+^ per Ca^2+^). Indirect experiments with isolated mitochondria under pathological conditions or Ca^2+^ overload suggest an additional, higher conductance route, through the transient opening of the mitochondrial permeability transition pore (mPTP). However, the physiological relevance of mPTP in Ca^2+^ homeostasis remains controversial and is not supported by genetic ablation studies (Ichas et al., 1997; Baines et al., 2005). In addition to its contribution in disease, which is discussed later, new roles for mitochondrial Ca^2+^ homeostasis are also emerging for normal neuron physiology. For example, it was recently described that olfactory sensory neurons require mitochondrial Ca^2+^ mobilization in order to encode intensity (Fluegge et al., 2012). Therefore, aberrant mitochondrial Ca^2+^ homeostasis in these neurons converts them into simple signal detectors and impairs their function in olfaction.

#### Calcium buffers and sensors

A large set of proteins with ability to bind Ca^2+^ specifically and reversibly provide yet another level of control in Ca^2+^ homeostasis by acting as sensors or buffers (**Figure 1**; **Table 1**). A large family of these Ca^2+^-binding proteins is the one containing EF-hand Ca^2+^ binding domains. These motifs consist of two 10–12 residue long alpha helices, oriented perpendicularly against each other, separated by a 12-residue long loop region. EF-hand domains often exist as multiple pairs generating a wide structural and functional variability within this large family of proteins (Kretsinger, 1980). A prominent member of this family, calmodulin, serves as a Ca^2+^ sensor that translates graded changes of intracellular Ca^2+^ concentration into a graded signaling response by interacting with various Ca^2+^-sensitive enzymes.

Another set of EF-hand-containing proteins, represented by calretinin, calbindin, and parvalbumin, function as Ca^2+^ buffers. These proteins are predominantly expressed by the inhibitory GABAergic interneurons of the central nervous system in specific patterns, therefore contributing to the diversification of these interneurons into distinct subtypes (Van Brederode et al., 1990). A multitude of studies has demonstrated that these proteins modulate the Ca^2+^ levels locally in the presynaptic active zone or at postsynaptic densities. Moreover, they are thought to actively and differentially participate in modulating neuronal vulnerability to different types of stress. In hippocampal primary cultures, neurons expressing calbindin are less vulnerable to oxidative stress-induced apoptosis because they recover Ca^2+^ concentration more effectively after stimulation, whereas in cortical neurons this is true for calretinin-containing neurons (Mattson et al., 1991). Similarly, genetic over-expression of parvalbumin in mice rescues motorneurons from injury-induced cell death (Dekkers et al., 2004).

It is generally thought that the transduction of the Ca^2+^ signal by EF-hand proteins consists a series of conformational changes that occur after Ca^2+^ has become bound. However, it is important to also mention that there are some exceptions, as no significant conformational changes after Ca^2+^ binding have been described for at least two of the EF-hand proteins, such as parvalbumin itself and calbindin, which are thus likely to act instead only as temporal Ca^2+^ buffers. Although most EF-hand proteins reside in the cytosol (and in the nucleoplasm), reticulocalbin is localized in the lumen of the ER (Tachikui et al., 1997). On the other hand, Cab45 (Scherer et al., 1996) and nucleobindin are localized in the Golgi apparatus (Lin et al., 1998) and glycerophosphate dehydrogenase (Pilstrom and Kiessling, 1972) and Aralar are located on the outer face of the inner mitochondrial membrane (del Arco and Satrustegui, 1998; Del Arco et al., 2000).

Another group of Ca^2+^-binding proteins, collectively known as intracellular neuronal calcium sensors (NCS; Braunewell and Gundelfinger, 1999; Burgoyne and Weiss, 2001), includes five subfamilies: the recoverins and guanylyl cyclase activating proteins (GCAPs), which are primarily expressed in retinal photoreceptor cells and have established roles in the regulation of photo-transduction; the frequenins, visinin-like and Kv-channel-interacting proteins (KChIPs), which are widely expressed in central neurons. One key feature of most NCS is N-terminal acylation: several members of the family are N-terminally myristoylated. Binding of Ca^2+^ to recoverin, and presumably to other NCS proteins, changes their conformation, exposing the myristoyl residue and hydrophobic portions of the molecule, making them available for membrane (or target protein) interaction. The Ca^2+^-myristoyl switch could be a mechanism that affects the compartmentation of signaling cascades in neurons and/or the transmission of Ca^2+^ signals to their membranes (Braunewell and Gundelfinger, 1999; Burgoyne and Weiss, 2001). Although the functions of the last three families are not clearly defined, it has been shown that they interact with multiple target proteins and with nucleic acids as well (Carrion et al., 1999). KChIP3 encodes the protein calsenilin, shown recently to interact with presenilin 1 and 2, two proteins whose mutations result in familial Alzheimer’s disease (AD; Buxbaum et al., 1998; Buxbaum, 2004). Relevant to the neurodegenerative phenotype of AD pathology, this interaction was shown to modulate the proteolytic processing of presenilins. In addition, two other NCS proteins, recoverin and GCAP1 have been involved in degenerative diseases of the retina. Mutations in the GCAP gene have been associated with autosomal dominant cone dystrophy. One of the defects has been related to constitutive activation of guanylyl cyclase that is not properly inactivated by high levels of Ca^2+^, characteristic of physiological dark conditions, eventually leading to degeneration of cone cells (Dizhoor et al., 1998; Sokal et al., 1998). The other condition [GCAP1(P50L); Sokal et al., 2000] is a milder form of autosomal dominant cone dystrophy in which the mutation reduces the Ca^2+^-binding ability of GCAP1. Recoverin has been identified as the autoantigen in a degenerative disease of the retina called cancer-associated retinopathy (CAR), in which patients lose vision due to degeneration of photoreceptors (Polans et al., 1991; Polans et al., 1995).

## BRAIN AGING AND THE “CALCIUM HYPOTHESIS”

The potential contribution of altered Ca^2+^ homeostasis at least to some aspects of brain aging and neurodegeneration was first put forward by Khachaturian in the 1980s, with the formulation of the “Ca^2+^ hypothesis of aging” (Gibson and Peterson, 1987; Disterhoft et al., 1994; Khachaturian, 1994). Early findings in the field that corroborated this hypothesis examined the major transport pathways of Ca^2+^ during aging and found that at least in some types of neurons, such as the principal cells in the hippocampal CA1 region, there is an increased Ca^2+^ influx mediated by increased VOCC activity in aged neurons (Landfield and Pitler, 1984; Thibault and Landfield, 1996). Similarly, Ca^2+^ extrusion through the PMCA was found to be decreased in aged neurons (Michaelis et al., 1996). Subsequently, the focus shifted toward the intracellular mechanisms of Ca^2+^ homeostasis and their deregulation during aging. Several studies demonstrated that there is an increased release of Ca^2+^ from the ER stores through both the InsP3 and RyR receptors (Thibault et al., 2007), leading to the proposal that release from the RyR receptor may be a useful biomarker of neuronal aging. Below, we will consider in more detail findings that relate to two key elements of aging: aberrant synaptic plasticity and neurodegeneration.

### ROLE OF CALCIUM IN SYNAPTIC PLASTICITY AND NEURONAL EXCITABILITY DURING AGING

Aging of the brain is manifested in humans by a progressive cognitive decline associated with weakening of the ability to process new information and of the executive function. The most dramatic effect is notably observed on the function of episodic memory, including spatial memory. The cognitive decline associated with normal aging is not attributed to significant neuronal loss (Gallagher et al., 1996), but is rather thought to result from changes in synaptic connectivity and plasticity. There is a general consensus that memory and learning are molecularly encoded by mechanisms controlling synaptic plasticity in several brain areas. Among these, the afferent pathways of the hippocampus are the most relevant, but other areas such as the amygdale, the visual, somatosensory and prefrontal cortices, and the subiculum also play important roles in processing, integration, and consolidation of new information. Using mainly the hippocampus, numerous studies have deciphered a major role for Ca^2+^ in the two major forms of synaptic plasticity, LTP (Bliss and Collingridge, 1993) and long-term depression (LTD). LTP represents an increase in synaptic transmission, induced by pattern stimulation of afferent fibers and it is the main process proposed to underlie memory formation. On the other hand, LTD is a means of decreasing synaptic strength, contributing to the loss of synaptic contacts and associated with increased forgetfulness during aging (Foster, 1999,^2007^; Zhou et al., 2004; Shinoda et al., 2005). Age-related changes in LTP and LTD underline the functional significance of altered synaptic plasticity for cognitive function (Foster and Norris, 1997; Foster, 1999; Foster and Kumar, 2002).

Relevant to the role of Ca^2+^ deregulation in memory loss, the critical event leading to induction of LTP appears to be the large influx of calcium ions into the postsynaptic spine. Importantly, LTP is blocked by injection of intracellular Ca^2+^ chelators such as EGTA (Lynch et al., 1983) or BAPTA (Mulkey and Malenka, 1992) and conversely, LTP is induced when the postsynaptic cell is loaded with calcium (Malenka et al., 1988). Therefore, it is well established that a significant elevation of postsynaptic Ca^2+^ concentration is both necessary and sufficient for the induction of hippocampal LTP (Bliss and Collingridge, 1993). In contrast, a modest rise in Ca^2+^ concentration results in induction of LTD through activation of protein phosphatases that dephosphorylate AMPA receptors (Artola and Singer, 1993; Lisman, 1989,^1994^). Due to the differential level of Ca^2+^ fluctuation involved in the generation of the various forms of synaptic plasticity, the stimulation patterns for the induction of LTP and LTD constitute high- and low-frequency stimulation, respectively.

In general, the effect of aging on synaptic plasticity can be summarized by several key observations: First, the threshold for induction of LTP increases such that higher stimulation frequencies or more induction sessions are required in older animals in order to achieve the same level of potentiation. Second, the threshold for induction of LTD is lowered in aged animals, facilitating its prevalence. Furthermore, the maintenance of LTP is disrupted such that the enhanced transmission decays more rapidly in aged animals. In contrast, LTD and depotentiation, or erasure of LTP, are increased in aged animals due to a lowering of the threshold stimulation needed for induction of synaptic depression (Norris et al., 1996; Foster and Norris, 1997; Kamal et al., 2000; Vouimba et al., 2000). Thus, the age-related decline in synaptic transmission (Barnes, 1994) may reflect a shift in the LTP/LTD balance, with insufficient LTP induction and maintenance and excessive synaptic depression (Foster et al., 2001).

In most of the synapses that support LTP (in the hippocampus and elsewhere), the postsynaptic increase in calcium is mediated through the activation of the NMDA receptor. As already mentioned earlier, NMDA receptor activation allows the influx of calcium only when the receptor is occupied by L-glutamate and concomitantly the postsynaptic membrane is depolarized. Emerging evidence indicates that the synaptic plasticity shift during aging results from changes in the source of Ca^2+^ such that Ca^2+^ influx through NMDARs is reduced (Lehohla et al., 2008; Bodhinathan et al., 2010) and Ca^2+^ influx through L-type VDCCs is increased (Barnes, 1994; Norris et al., 1996; Thibault and Landfield, 1996; Shankar et al., 1998; Potier et al., 2000). The increase could arise from altered gene or protein expression (Herman et al., 1998), or phosphorylation changes of the L-type Ca^2+^ channels (Norris et al., 2002; Davare and Hell, 2003). Interestingly, the L-type Ca^2+^ channel blocker nimodipine counteracts age-related learning impairment in rabbits (Deyo et al., 1989; Kowalska and Disterhoft, 1994), rodents (Levere and Walker, 1992), non-human primates (Sandin et al., 1990), and elderly patients with dementia (Ban et al., 1990; Tollefson, 1990).

Additionally, aged neurons show a multitude of defects in Ca^2+^ homeostasis, including enhanced release of Ca^2+^ from the ER (Kumar and Foster, 2004; Gant et al., 2006), diminished Ca^2+^ extrusion through the plasma membrane ATPase (Michaelis et al., 1996; Gao et al., 1998), reduced cellular Ca^2+^ buffering capacity due to impairment of the SERCA pumps (Murchison and Griffith, 1999), and diminished mitochondrial Ca^2+^ sink capability (Murchison and Griffith, 1999; Xiong et al., 2002). The overall result is an increase of Ca^2+^ loads which negatively impact neuronal excitability (Landfield and Pitler, 1984; Khachaturian, 1989; Matthews et al., 2009). Moreover, such an increase in intracellular Ca^2+^ concentration increases the threshold frequency for induction of LTP (Shankar et al., 1998; Ris and Godaux, 2007), and enhances the susceptibility to induction of LTD (Norris et al., 1996; Kumar and Foster, 2005), ultimately explaining the age-associated deficits in learning and memory. In line with this notion, administration of the cell permeable Ca^2+^ chelator BAPTA, ameliorates impaired presynaptic cytosolic and mitochondrial Ca^2+^ dynamics in hippocampal CA1 synapses of old rats (Tonkikh and Carlen, 2009), and enhances spatial learning (Tonkikh et al., 2006).

In the context of LTP induction, a key early finding was the observation that postsynaptic entry of calcium leads to activation of Ca^2+^/calmodulin complex-dependent kinase II (CaMKII), one of the most abundant proteins in neurons comprising 1–2% of the total protein. Although it is expressed both pre- and postsynaptically, its expression is particularly high in the postsynaptic density, where it is ideally located to respond to changes in calcium concentration. There are more than 30 isoforms of CaMKII and numerous substrates, many of which are located in the postsynaptic density (Fink and Meyer, 2002). CaMKII is generally considered a mediator of primary importance in linking transient calcium signals to neuronal plasticity. Importantly, observations by Silva et al. (1992a),b,(c) indicated that deletion of the CaMKII gene in mice results in impaired LTP and aberrant spatial memory. Moreover, activation of CaMKII is significantly reduced in aged hippocampal neurons (Mullany et al., 1996). The data obtained from studies on rodents have to a large extent, been paralleled by similar findings in other organisms, indicating that several models expressing various forms of synaptic plasticity exhibit a requirement for CaMKII activation. For instance, CaMKII knockout in *Drosophila* exhibits impaired associative learning, while motor and sensory systems remain unaffected (Joiner and Griffith, 1999). Similarly, knockout of *unc-43* (a gene encoding the CaMKII analog in *C. elegans*) affects the stability of synapses and general neuronal physiology, ultimately culminating in altered function of olfactory neurons (Sagasti et al., 2001).

Beyond activating the CaMKII signaling cascade, Ca^2+^ also acts as a second messenger that is responsible for the activity-dependent transcription of several key genes (West et al., 2001). The products of these genes are necessary in order to convert the effects of transient stimuli into long-term changes in brain function, a process that is required for the formation of memories. Of the neural-selective activity-dependent genes, brain-derived neurotrophic factor (BDNF) is activated by calcium influx through L-type VOCCs (L-VOCCs) acting on the transcription of *BDNF* from promoter III (West et al., 2001). BDNF is among the most relevant calcium targets for the modulation of memory. BDNF transcription is up-regulated dramatically by membrane depolarization *in vitro* (Ghosh et al., 1994; Tao et al., 1998) and by induction of LTP, and associative learning (Ernfors et al., 1991; Patterson et al., 1992; Tokuyama et al., 2000). Moreover, loss of BDNF is associated with impaired LTP among other synaptic defects. It is also well established that BDNF transcription is largely decreased during aging (Tapia-Arancibia et al., 2008), and that epigenetic induction of BDNF transcription in aged subjects significantly ameliorates the cognitive and memory defects associated with aging (Zeng et al., 2011). A summary of the perturbations of Ca^2+^ homeostasis associated with nervous system aging is shown in **Table 2**.

**Table 2:** Perturbations of Ca^**2**+^ homeostasis in the aging nervous system.

ca^2+^ deregulation associated with aging of the nervous system	Reference
Increased ca^2+^ influx mediated by voltage-dependent calcium channels	Landfield and Pitler (1984), Thibault and Landfield (1996)
Decreased ca^2+^ extrusion through the plasma membrane pump (PMCA)	Michaelis et al. (1996), Gao et al. (1998)
Increased release of ca^2+^ from the ER stores through both the InsP3 and RyR receptors	Thibault et al. (2007)
Reduced ca^2+^ influx through NMDARs	Lehohla et al. (2008), Bodhinathan et al. (2010)
Increased ca^2+^ influx through L-type VDCCs	Barnes (1994), Norris et al. (1996), Thibault and Landfield (1996), Shankar et al. (1998), Potier et al. (2000)
Phosphorylation changes of the L-type ca^2+^ channels	Norris et al. (2002), Davare and Hell (2003)
Increased release of ca^2+^ from the ER	Gant et al. (2006), Kumar and Foster (2004)
Impairment of the SERCA pumps	Murchison and Griffith (1999)
Diminished mitochondrial ca^2+^ sink capability	Murchison and Griffith (1999), Xiong et al. (2002)
Reduced activation of CaMKII in hippocampal neurons	Mullany et al. (1996)
Reduced ca^2+^-dependent transcription of genes such as BDNF	Tapia-Arancibia et al. (2008)

### ROLE OF CALCIUM IN AGING-RELATED NEURODEGENERATION

Aging is the greatest risk factor for the development of neurodegenerative disorders. These include a diverse collection of pathologies characterized by the late onset and gradual loss of specific neuronal subpopulations in motor, sensory, or cognitive systems. Despite major intrinsic differences in the etiology of each disorder, deregulated Ca^2+^ homeostasis has emerged as a common underlying mechanism of neuronal loss in AD, Parkinson’s (PD) diseases, amyotrophic lateral sclerosis (ALS), and other neurodegenerative disorders (Mattson, 2007; Bezprozvanny, 2009).

Alterations of Ca^2+^ homeostasis may be in some cases directly responsible for neuronal death. Persistently increased levels of intracellular Ca^2+^ can result in severe phenotypes in neurons, culminating to neuronal death and degeneration (Siman et al., 1989; Celsi et al., 2009). This process is often specifically mediated or even initiated by the diminished capacity of mitochondria to buffer Ca^2+^. An example where there is ample evidence that altered mitochondrial Ca^2+^ homeostasis mediates neuronal loss is ALS, an adult onset disease, with incidence increasing with age. ALS is characterized by selective and progressive degeneration of motorneurons in the spinal cord and brain, leading to weakness, atrophy, and paralysis of voluntary muscles. Mutations in superoxide dismutase (SOD1) are the most common genetic factors responsible for about 20% of familial ALS cases (Rosen et al., 1993). SOD1 is a ubiquitously expressed enzyme that converts superoxide to hydrogen peroxide in order to protect cells against oxidative stress. While there is still no consensus as to how mutant SOD1 causes selective toxicity to motorneurons, increasing evidence suggests that the mechanisms largely concentrate on the dysfunction of ER and mitochondrial Ca^2+^ homeostasis (Bacman et al., 2006; Hervias et al., 2006; Magrane et al., 2009; Shi et al., 2010).

At the level of the ER, a recent paper implicates the Ca^2+^ buffering protein calreticulin in the death of motorneurons in a model of ALS (Bernard-Marissal et al., 2012). More specifically, fast fatigable motorneurons selectively activate an ER stress response that drives their early degeneration, while a subset of mSOD1 motorneurons shows exacerbated sensitivity to activation of the motorneuron-specific Fas (transmembrane TNF receptor superfamily member 6) and nitric oxide (NO) pathway. However, the links between the two mechanisms and the molecular basis of their cellular specificity remained unclear. This paper demonstrates that Fas activation causes reduced levels of calreticulin specifically in mSOD1 motorneurons. Decreased expression of calreticulin is both necessary and sufficient to trigger SOD1(G93A) motorneuron death through the Fas/NO signaling pathway, and represents an early event that precedes muscle denervation and is restricted to vulnerable motor pools.

At the mitochondrial level, altered Ca^2+^ handling also appears early on, before motorneuron degeneration is manifested, suggesting that it is actively involved in disease pathogenesis. SOD1, which is a predominantly cytosolic protein, also localizes to the ER and mitochondria (Jaarsma et al., 2001; Okado-Matsumoto and Fridovich, 2001; Higgins et al., 2002; Mattiazzi et al., 2002), predominantly in the intermembrane space and less so on the outer membrane (Pasinelli et al., 2004; Vande Velde et al., 2008) and matrix (Vijayvergiya et al., 2005). By mechanisms that are still poorly understood, mutant SOD1 induces increased Ca^2+^ uptake by mitochondria, as convincingly demonstrated in mitochondria isolated from the brain and spinal cord of SOD1 mutant mice (Damiano et al., 2006). This defect appears to be neuron-specific, as liver cells from the same mutants retain unaffected mitochondrial Ca^2+^ homeostasis. Impaired Ca^2+^ handling by mitochondria is thought to be the primary cause of the abnormally high concentration of intracellular Ca^2+^ observed in ALS motorneurons (Carri et al., 1997; Kruman et al., 1999), making them vulnerable to degeneration (Kim et al., 2002,^2007^).

Mitochondrial Ca^2+^ overload is associated with activation of cell death pathways (Bernardi et al., 1999) and is observed in many pathological conditions in addition to ALS (Honda and Ping, 2006; Norenberg and Rao, 2007). The mechanisms responsible for Ca^2+^ overload are not entirely clear; however, their elucidation could provide a base for significant pharmacological interventions in the future. Theoretically, defects of the mitochondrial NCX could be involved in causing Ca^2+^ overload in ALS, although this putative mechanism remains to be directly explored. Another potential factor contributing to Ca^2+^ overload could be the functional and physical link between mitochondria and ER. Transfer of Ca^2+^ from the large stores in the ER to mitochondria depends on the relative positioning of these two organelles, and it is thought to occur at Ca^2+^ “hotspots”, sites where ER and mitochondrial membranes are in close physical contact (Rizzuto et al., 1999). Shortening the distance between the two organelles was shown to result in increased accumulation of Ca^2+^ in mitochondria, causing cell death (Csordas et al., 2006). Since mutant SOD1 accumulates both in ER (Kikuchi et al., 2006; Urushitani et al., 2006) and mitochondrial (Liu et al., 2004) membranes, it is plausible that the structure of these calcium hotspots is altered in mutant neurons, leading to abnormal handling of Ca^2+^ between the two organelles.

Whatever the mechanism of the increased Ca^2+^ accumulation in mitochondria, activation of cell death by mitochondrial Ca^2+^ overload involves the opening of the mPTP, followed by release of cytochrome *c*, and downstream activation of apoptosis. Cytochrome *c* released into the cytosol can further propagate apoptotic signaling by binding to the IP3-R on the ER, desensitizing its autoinhibition by calcium and thus causing further calcium release from ER stores (Boehning et al., 2003). Ablation of cyclophilin D (CypD), a modulatory component of the mPTP, delays the opening of mPTP (Basso et al., 2005) and has a protective effect against neuronal death in models of ischemia (Baines et al., 2005; Schinzel et al., 2005). In ALS, it was also reported that loss of CypD in SOD1 mutant mice delays the onset of the disease and significantly extends lifespan (Martin et al., 2009). Moreover, two studies using the immunosuppressant cyclosporin A, which binds to CypD to inhibit mPTP, in mutant SOD1 mice, suggest that inhibition of mPTP may be of benefit to ALS (Keep et al., 2001; Kirkinezos et al., 2004).

Another mechanism whereby Ca^2+^ contributes to the activation of cell death is by stimulating the production of mitochondrial reactive oxygen species (ROS). Oxidative stress caused by the damaging effect of ROS to proteins, lipids, and DNA, is a common feature of aging-related diseases, including ALS (Floyd and Hensley, 2002; Lin and Beal, 2006). Mitochondrial dysfunction (Wei, 1998), and particularly mitochondrial Ca^2+^ overload (Petrosillo et al., 2004), increases ROS production. In particular, increased levels of mitochondrial Ca^2+^ enhance cytochrome *c* release through a mechanism involving ROS-mediated oxidation of cardiolipin (Vercesi et al., 1997; Iverson and Orrenius, 2004). Notably, lipid peroxidation (Mattiazzi et al., 2002) and dissociation of cytochrome *c* from the mitochondrial inner membrane (Kirkinezos et al., 2005) have been reported in mutant SOD1 mice, but also in PD (Beal, 2003), and AD (Green and Kroemer, 2004;Lin and Beal, 2006; Kawamoto et al., 2012; Lee et al., 2012a).

Alzheimer’s disease is perhaps the most widespread neurodegenerative disorder of the elderly, with most familiar cases attributed to several mutations in presenilin 1 and 2, genes whose protein products are responsible for the proteolytic cleavage of the amyloid precursor peptide (APP). The mechanism by which presenilin mutations cause AD involves increased production of Aβ1–42 which aggregates and damages neurons. This view has been recently expanded by emerging findings suggesting that perturbed ER Ca^2+^ homeostasis significantly contributes to the dysfunction and degeneration of neurons in AD (Kipanyula et al., 2012). For example, recent work indicates that there is impaired Ca^2+^ uptake by mitochondria in the dentate gyrus of a mouse model of AD (Lee et al., 2012b). This can be explained to some extent by the novel role proposed by at least two groups for presenilins as regulators of Ca^2+^ homeostasis in the ER (Pack-Chung et al., 2000; Yoo et al., 2000). Interestingly, mutations in presenilin 1 that cause early onset familial AD, increase the pool of ER Ca^2+^ available for release, and enhance Ca^2+^ release from the ER through IP3- and RyR receptors (Chan et al., 2000; Guo et al., 1996,^1999^; Cheung et al., 2010; Leissring et al., 2000). Future research should clarify the specific contributions of perturbed ER Ca^2+^ handling to the cellular events that underlie synaptic dysfunction and neuronal degeneration in AD. While elevated pools of ER Ca^2+^ resulting from mutations in presenilins have been widely documented in a range of cell culture and animal models, the molecular basis of this alteration remains unknown and is potentially a key field for the development of novel pharmacological targets.

In addition to direct effects on neuronal survival, altered Ca^2+^ homeostasis is also likely to contribute to the initiation or progression of the neurodegenerative process by enhancing neuronal vulnerability to metabolic and other stressors (Toescu and Verkhratsky, 2004; Toescu and Vreugdenhil, 2010). One such example is the population of basal forebrain cholinergic neurons, a group of neurons that are selectively vulnerable to pathology and loss early in AD, as well as in a number of other neurodegenerative disorders of the elderly. In the primate, including man, these neurons are rich in the Ca^2+^ buffer protein calbindin. Notably, there is a substantial loss of calbindin in the course of normal aging and a further loss in AD(Iacopino and Christakos, 1990). Significantly, cholinergic neurons that had lost their calbindin in the course of normal aging were those that selectively degenerated in AD, while calbindin-containing neighboring neurons were virtually resistant to the process of tangle formation, a hallmark of the disease (Riascos et al., 2011). Another study reported that over-expression of calbindin in presenilin 1 mutant neurons was sufficient to prevent apoptosis (Guo et al., 1998). Similarly, a dramatic reduction in the Ca^2+^ buffering protein calbindin levels has been described in brains of PD patients (Iacopino and Christakos, 1990) and dopaminergic (DA) neurons expressing higher levels of calbindin, or other Ca^2+^ buffers such as calretinin and parvalbumin, were shown to be resistant to degeneration in PD (Yamada et al., 1990; Tsuboi et al., 2000). These findings are consistent with earlier findings suggesting that calbindin-positive hippocampal neurons are more resistant against oxidative stress (Mattson et al., 1991), although other Ca^2+^ buffer proteins seem to confer resistance to stress in different neuronal subpopulations. Understanding the mechanisms underlying such an instructive function of Ca^2+^ buffer proteins is of great importance as there may be a yet unidentified crosstalk with major signaling cascades. More work in this direction would greatly enhance our ability to selectively intervene in order to modulate the vulnerability of distinct neuronal populations.

Similar to ALS and AD, PD is another case where Ca^2+^ deregulation has recently attracted a lot of attention. PD is characterized by motor defects resulting from the selective loss of DA neurons in the substantia nigra and intracellular accumulation of cell aggregates known as Lewy bodies, mostly composed of α-synuclein. The idea that mitochondria could be directly involved in the pathogenesis of PD comes from the early accidental observation that 1-methyl-4-phenyl-1,2,3,6-tetrahydropyridine (MPTP), an inhibitor of the mitochondrial respiratory chain complex I, causes Parkinson-like symptoms (Langston and Ballard, 1983). Later on, it was also demonstrated that DA neurons from PD patients show massive accumulation of mitochondrial DNA (mtDNA) deletions that impair the function of the respiratory chain complexes (Exner et al., 2012), thus increasing the probability of dysfunctions in these organelles.

Some clues as to the selective vulnerability of this population arise from the fact that DA neurons of the substantia nigra display unusual physiological properties. First, unlike most other neurons in the brain, they are autonomously active, generating regular action potentials in the absence of synaptic input (Grace and Bunney, 1983). This pacemaking activity is thought to maintain physiological levels of dopamine in regions they innervate, particularly the striatum (Romo and Schultz, 1990). To drive this pacemaking activity, these neurons rely, at least in part, on a rare form of L-type Ca^2+^ channels (Bonci et al., 1998; Ping and Shepard, 1996; Puopolo et al., 2007) comprised of the Cav1.3 pore-forming subunit (Striessnig et al., 2006; Chan et al., 2007). This leads to typically elevated intracellular Ca^2+^ concentrations under physiological conditions (Wilson and Callaway, 2000; Chan et al., 2007). Second, DA neurons of the substantia nigra display an elaborate axonal network (Matsuda et al., 2009), supporting orders of magnitude more synapses compared to a cortical pyramidal neuron (Arbuthnott and Wickens, 2007). As a result, the mitochondrial density in their somatic and dendritic regions is very low compared to other neuronal types (Liang et al., 2007). Taken together, these characteristics are thought to contribute to an intrinsic state of increased metabolic stress, where increased load of intracellular Ca^2+^ is met by a depleted mitochondrial network.

Additional genetic factors could increase the rate at which mitochondrial Ca^2+^ homeostasis is compromised in these already vulnerable neurons. At least 13 gene loci and 9 genes have been linked to both autosomal dominant and recessive forms of PD (Lesage and Brice, 2009). Mutations in three proteins encoded by these genes, namely, parkin (PARK2), DJ-1 (PARK7), and PINK1 (PARK_6_), are associated with recessive early onset forms of PD, whereas mutations in α-synuclein (PARK1–4) and LRRK2 (PARK_8_) are responsible for dominant forms of familial PD. Mitochondrial dysfunction has been described for mutants of all these genes (Lesage and Brice, 2009).

Recent papers have started to explore in more detail the possibility of Ca^2+^ handling by the PD-related proteins. DJ-1 is a multitask protein that, in addition to its main role as an antioxidant (Taira et al., 2004), is also involved in maintaining cytosolic basal Ca^2+^ concentration values to permit depolarization-induced Ca^2+^ release from the sarcoplasmic reticulum in muscle cells (Shtifman et al., 2011). Moreover, DJ-1 was shown to protect DA neurons from Ca^2+^-induced mitochondrial uncoupling and ROS production during physiological pacemaking (Guzman et al., 2010).

Regarding α-synuclein, it has been described that it can modulate Ca^2+^ influx from the extracellular milieu by enhancing the plasma membrane ion permeability (Danzer et al., 2007) either through their direct insertion into the plasma membrane and the formation of a pore (Lashuel et al., 2002) or through the modulation of plasma membrane Ca^2+^ permeability (Furukawa et al., 2006). The actual mechanisms through which α-synuclein aggregation and Ca^2+^ dysfunction influence each other are not clear, however, a functional interplay is unambiguous: Increased intracellular Ca^2+^ promotes α-synuclein aggregation, which in turn could promote intracellular Ca^2+^ increase (Nath et al., 2011). A recent study suggests that using its C-terminal domain, α-synuclein controls mitochondrial calcium homeostasis by enhancing ER–mitochondria interactions (Cali et al., 2012). As these results were obtained *in vitro* using non-neuronal cell lines, their relevance to DA neuron physiology and pathology remains to be examined.

As to PINK1, its direct role in regulating cellular, and most specifically mitochondrial Ca^2+^ fluxes, has been recently proposed starting with the observation that the co-expression of mutant PINK1 in a cellular model of PD-expressing mutated α-synuclein exacerbated the observed mitochondrial defects, that is, increased mitochondrial size with loss of cristae and reduced ATP levels (Marongiu et al., 2009). The proposed mechanisms of PINK1 action was based on a deregulation of mitochondrial Ca^2+^ influx. As by blocking mitochondrial Ca^2+^ uptake, it was possible to restore the original phenotype (Marongiu et al., 2009), thus suggesting that mutant PINK1 could reinforce α-synuclein pathology by acting on converging pathways affecting mitochondrial function. Other studies have further investigated the role of PINK1 in mitochondrial Ca^2+^ metabolism, but the results are controversial. In one case, it was proposed that PINK1 absence caused an impairment of mitochondrial Ca^2+^ efflux, probably affecting the mitochondrial Na^+^/Ca^2+^ exchanger activity and thus resulting in mitochondrial Ca^2+^ overload, ROS production, and impaired respiration (Gandhi et al., 2009). In another very recent study, PINK1 depletion has instead been shown to impair mitochondrial Ca^2+^ uptake and consequently to affect energy metabolism (Heeman et al., 2011). However, consistently, numerous reports showed that PINK1-deficient cells have impaired mitochondrial membrane potential and enhanced sensitivity to the toxic effects of mitochondrial complex I inhibitors (Wood-Kaczmar et al., 2008), as well as enhanced Ca^2+^ vulnerability (Akundi et al., 2011).

## OUTLOOK

Given the fundamental importance of Ca^2+^ homeostasis in the biology of all cells, it is not completely surprising that more and more studies suggest that deregulated Ca^2+^ is actively involved in the course of normal aging and in diverse pathological conditions. A general message arising from these studies is that in the nervous system Ca^2+^ signaling and homeostasis should be examined in view of the amazing cellular diversity exhibited by the nervous system. The machinery controlling Ca^2+^ homeostasis is similarly diverse among neurons, uniquely suited to the needs of each neuronal subtype. Taken together, the intrinsic differences of neurons in morphology, connectivity, proteome and Ca^2+^ homeostatic machinery are very likely to collectively and synergistically contribute to the selective vulnerability of distinct neuronal populations to different causes of senescence. The more we understand the interplay of Ca^2+^ homeostatic mechanisms with the intrinsic qualities of different neurons, the closer we will get to developing cell-specific therapies.

## Conflict of Interest Statement

The authors declare that the research was conducted in the absence of any commercial or financial relationships that could be construed as a potentialconflict of interest.
